# Development of leafhopper cell culture to trace the early infection process of a nucleorhabdovirus, rice yellow stunt virus, in insect vector cells

**DOI:** 10.1186/s12985-018-0987-6

**Published:** 2018-04-20

**Authors:** Haitao Wang, Juan Wang, Yunjie Xie, Zhijun Fu, Taiyun Wei, Xiao-Feng Zhang

**Affiliations:** Fujian Province Key Laboratory of Plant Virology, Institute of Plant Virology, Fujian Agriculture and Forestry University, Fuzhou, People’s Republic of China

**Keywords:** *Nucleorhabdovirus*, Rice yellow stunt virus, Nucleoprotein and phosphoprotein, Viroplasm-like structures, Insect vector

## Abstract

**Background:**

In China, the rice pathogen *Rice yellow stunt virus* (RYSV), a member of the genus *Nucleorhabdovirus* in the family *Rhabdoviridae*, was a severe threat to rice production during the1960s and1970s. Fundamental aspects of the biology of this virus such as protein localization and formation of the RYSV viroplasm during infection of insect vector cells are largely unexplored. The specific role(s) of the structural proteins nucleoprotein (N) and phosphoprotein (P) in the assembly of the viroplasm during RYSV infection in insect vector is also unclear.

**Methods:**

In present study, we used continuous leafhopper cell culture, immunocytochemical techniques, and transmission electron microscopy to investigate the subcellular distributions of N and P during RYSV infection. Both GST pull-down assay and yeast two-hybrid assay were used to assess the in vitro interaction of N and P. The dsRNA interference assay was performed to study the functional roles of N and P in the assembly of RYSV viroplasm.

**Results:**

Here we demonstrated that N and P colocalized in the nucleus of RYSV-infected *Nephotettix cincticeps* cell and formed viroplasm-like structures (VpLSs). The transiently expressed N and P are sufficient to form VpLSs in the Sf9 cells. In addition, the interactions of N/P, N/N and P/P were confirmed in vitro. More interestingly, the accumulation of RYSV was significantly reduced when the transcription of N gene or P gene was knocked down by dsRNA treatment.

**Conclusions:**

In summary, our results suggest that N and P are the main viral factors responsible for the formation of viroplasm in RYSV-infected insect cells. Early during RYSV infection in the insect vector, N and P interacted with each other in the nucleus to form viroplasm-like structures, which are essential for the infection of RYSV.

**Electronic supplementary material:**

The online version of this article (10.1186/s12985-018-0987-6) contains supplementary material, which is available to authorized users.

## Background

Plant negative-strand RNA viruses, such as rhabdoviruses, tenuiviruses, emaraviruses, and bunyaviruses are transmitted by their respective insect vectors in a persistent-propagative manner [1–3]. Plant rhabdoviruses have been taxonomically divided into four genera, *Cytorhabdovirus*, *Nucleorhabdovirus*, *Dichorhavirus,* and *Varicosavirus* [4]. Nucleorhabdoviruses, such as rice yellow stunt viru*s* (RYSV), sonchus yellow net virus (SYNV), potato yellow dwarf virus (PYDV), and maize mosaic virus (MMV), replicate and assemble within viral inclusions called viroplasms in the nucleus of their plant host or insect vector cells [5]. Currently, the mechanisms responsible for the genesis and maturation of the viroplasm induced by plant rhabdoviruses remain largely unknown.

The replication strategy of SYNV serving as paradigm for the cell biology of plant-adapted rhabdoviruses, has been studied extensively. The structural proteins N and P of SYNV in plant cells have been verified to contain nuclear localization signals (NLSs) and to be responsible for the formation of viroplasm in the nucleus [6, 7]. The viroplasm induced by SYNV accumulates in the perinuclear space of the infected plant cells under the participation of the N and P proteins, which is spatially separated from the site of virion assembly [8, 9]. Despite excellent investigations on the cell biology of plant-adapted rhabdoviruses in plants [6–10], very little progress has been made on their insect vectors. Continuous cell cultures of insects are uniquely suited to the investigation of virus infection because the early stages of viral infection in their insect vectors can be traced and a uniform viral infection can be maintained [11–15]. In this study, we used continuous cell cultures of *Nephotettix cincticeps*, one of the insect vectors of RYSV, to study the assembly and the accumulation site of RYSV in the insect cells. Knowledge on the formation of viroplasms of plant rhabdoviruses in its insect vector will further our understanding of nucleorhabdovirus biology and may lead to new strategies to control the vector-borne plant diseases.

Rice yellow stunt disease was first reported in 1965 in Taiwan and southern China [16, 17] and caused great losses in rice yields in southern China from the 1970s to 1980s. The causal pathogen, RYSV has a nonsegmented, negative single-stranded RNA genome, which contains seven genes in the order 3’-*N*-*P*-*3*-*M*-*G*-*6*-*L*-5′ [18, 19], encoding the nucleoprotein (N), phosphoprotein (P), matrix protein (M), glycoprotein (G), large RNA polymerase (L), putative movement protein, P3 and RNA silencing suppressor P6 [20–25]. The N and P proteins are essential nucleocapsid core components in all plant rhabdoviruses. Currently, we know little about the specific roles and relationships of the structural proteins N and P during RYSV infection of the insect vector cells. In addition, whether N and P are related to the formation of viroplasm in its insect vector is still unknown.

Here, we used immunocytochemical techniques, leafhopper vector cultured cells in monolayers (VCMs), and RNA interference (RNAi) assay to explore the roles of N and P of RYSV in its insect vector. Our results suggest that RYSV N co-localizes and interacts with P, and both proteins contribute to the formation of VpLSs in the nucleus. In addition, the knock down of either N or P gene strongly inhibited the infection of RYSV in its insect cells.

## Materials and methods

### Insects, cell culture and reagents

Nonviruliferous leafhoppers (*N. cincticeps*) were collected from Yunnan Province in southern China and reared on rice seedlings in cages in a controlled environment at 28 °C with 75 ± 5% humidity and 16 h light/8 h dark cycle. RYSV-infected rice samples were propagated via transmission by *N. cincticeps*. Continuous monolayer cultures of vector cells were originally developed from embryonic fragments of *N. cincticeps* maintained in LBM growth medium at 25 °C as described previously [26]. When the cells were cultured on coverslips and reached 80% confluence, the cells were washed with a solution of 0.1 M histidine that contained 0.01 M MgCl_2_ (pH 6.2) (His-Mg) and then inoculated with 50 μl viral inoculum prepared from RYSV infected rice leaves as previously described [27]. Cells were incubated for 2 h and then washed with His-Mg, and covered with growth medium before fixation. Rabbit/mouse polyclonal antisera against N, P, and virion were prepared as previously described [14, 15]. Virion antibody specifically detected RYSV structural proteins including N, P, M and G. IgGs were purified from the respective protein-specific polyclonal antibodies and conjugated directly to fluorescein isothiocyanate (FITC), rhodamine or Alexa Fluor 633 (Invitrogen) according to the manufacturer’s instructions.

### Immunofluorescence microscopy

RYSV-infected VCMs or the model cell line *Spodoptera frugiperda* (Sf9) infected with recombinant baculoviruses grown on glass coverslips were fixed at different times after inoculation in 4% *v*/v paraformaldehyde in 0.01 M phosphate-buffered saline buffer (PBS) at room temperature for 2 h and permeabilized at room temperature in 0.2% Triton X-100 in 0.01 M PBS buffer for 30 min. Cells were then incubated with a 100-fold-diluted solution of the directly conjugated IgG. Samples were visualized with a Leica TCS SP5 inverted confocal microscope, as described previously [28].

### Baculovirus expression of RYSV N and P proteins

A recombinant baculovirus expression system was used to study the localization of RYSV proteins, N and P in *Spodoptera frugiperda* (Sf9) cells, as described previously [29]. The baculovirus recombinant vectors expressing N fused with 6 × His tag (N-His) or P fused with a Strep tag (P-Strep) were used to transform *Escherichia coli* DH10 Bac cells (Invitrogen), respectively. Sf9 cells were transfected with the recombinant bacmids using Cellfectin II reagent (Thermo Fisher Scientific, USA) according to the manufacturer’s instructions. The recombinant baculovirus and healthy Sf9 cells were then examined using immunofluorescence microscopy and electron microscopy at different time points post inoculation [29].

### Transmission electron microscopy

The head tissues of RYSV-infected (46 sections) and healthy *N. cincticeps* were dissected, fixed, dehydrated and embedded, as described previously [30]. The ultrathin sections of VCMs prepared with an ultramicrotome (Leica UC7) were incubated with N-specific or P-specific IgGs, then subjected to immunogold labeling with goat antibodies against rabbit IgG conjugated with 15-nm gold particles or goat antibodies against mouse IgG conjugated with 10-nm gold particles (Sigma), as previously described [30], and examined with an electron microscope.

### Yeast two-hybrid assay

To detect the interaction between N and P, N and N as well as P and P, we used a yeast two-hybrid assay and the Matchmaker Gal4 Two-Hybrid System 3 (Clontech). The full-length N and P gene of RYSV were each amplified and respectively cloned into the pGBKT7 bait vector and pGADT7 prey vector. The bait and prey plasmids (pGBKT7-N/pGADT7-P, pGBKT7-N/pGADT7-N, and pGBKT7-P/pGADT7-P) were used to co-transform the AH109 yeast strain, and β-galactosidase activity was detected on SD/−Leu/−Trp/-His/−Ade/X-a-gal culture medium. The positive control (pGBKT7–53/pGADT7-T) and negative controls (pGBKT7-Lam/pGADT7-T, pGBKT7-N/pGADT7 and pGBKT7/pGADT7-P) were transformed in the same way.

### GST pull-down assay

The GST pull-down assay was used to detect any interaction of N with P, N with N, as well as P with P of RYSV. The N gene was amplified and cloned into pGEX-3X vector, which included a GST-tag (GST-N). The RYSV P gene was cloned and inserted into the His-fused vector pDEST17 (His-P). The P gene was also cloned into the pGEX-3X vector (GST-P), and the N gene was cloned into pDEST17 (His-N). The constructed plasmids, pDEST17-P/N, pGEX-3X-N/P and pGEX-3X (GST), were respectively expressed in *E. coli* strain BL21. Lysates of GST-N/GST-P and GST were incubated with glutathione-Sepharose beads for 3 h and then incubated with the lysates of His-N or His-P for 4 h, respectively. Finally, the mixtures were washed with elution buffer and detected with western blotting using GST-tagged and His-tagged antibodies (Abcam), respectively.

### Knockdown of N and P gene transcription by RNA interference induced by in vitro synthesized dsRNAs on viral infection in VCMs

The double strands RNA of N (*dsN*), P (*dsP*) and GFP (*dsGFP*) gene were synthesized according to the manufacturer’s instructions as previously described [31, 32]. VCMs were transfected with double strands RNAs (1 μg), *dsN*, *dsP* and *dsGFP* via the Cellfectin II reagent, respectively, for 8 h and then incubated with RYSV inoculum for 2 h [27, 30]. Infected cells were fixed at 72 hpi, immunolabeled with the specific antibody against N or P and then examined with immunofluorescence microscopy. The accumulation of N and P was analyzed with western blotting using N-specific (826 mg/mL, 1:2000) or P-specific (846 mg/mL, 1:2000) IgGs, respectively. RT-qPCR was also used to detect the transcript level of the N, P and M genes (primers are described in Additional file1) to verify the results of RNAi and the infection of RYSV. Three biological repeats were used for the RT-qPCR and analyzed using Student’s *t*-test.

## Results

### Morphogenesis of RYSV viral particles associated with the viroplasm in infected vector *N. cincticeps*

**Fig. 1:**
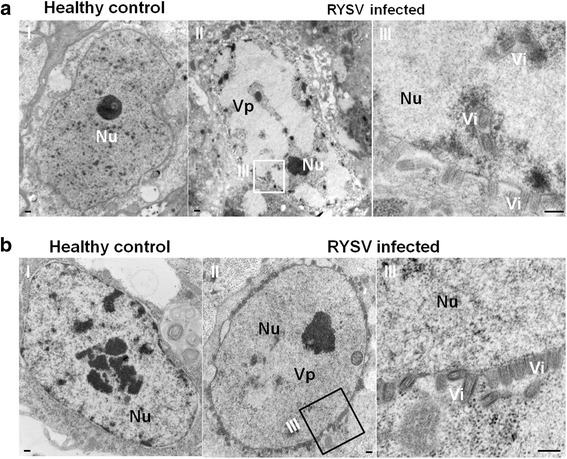
Morphogenesis of RYSV viral particles associated with the viroplasm matrix in the insect vector *N. cincticeps*. **a** Viroplasm matrix in the nucleus of cells from the head tissue of *N. cincticeps* after RYSV infection. **a-I** Cell of healthy *N. cincticeps*. Panel III is an enlargement of the boxed area in II (RYSV-infected *N. cincticeps*). **b** VCMs infected by RYSV, with virions around the nucleus but not in the control (I). Nu, nucleus; Vi, virion (s); Vp, viroplasm. Bars, 100 nm

In ultrathin sections of head tissues of RYSV-infected *N. cincticeps*, The electron-lucent VpLSs, apart from the chromatin and nucleolus, was located in the nucleus and filled most of the nucleus volume, comparing with the healthy control (Fig. 1a I-II). Most mature virions were observed in arrays around the nucleus and in vesiculations from the inner and the outer membranes of the nuclear envelope to the membranes surface, to a lesser extent, virions aggregated in a crystalline array in the expanded perinuclear space (Fig. 1a III). Similar results were observed in ultrathin sections of RYSV-infected VCMs (Fig. 1b II-III).

### Immunoelectron microscopy and immunofluorescence microscopy analysis of subcellular localization of N and P protein in RYSV-infected *N. cincticeps* cells

**Fig. 2:**
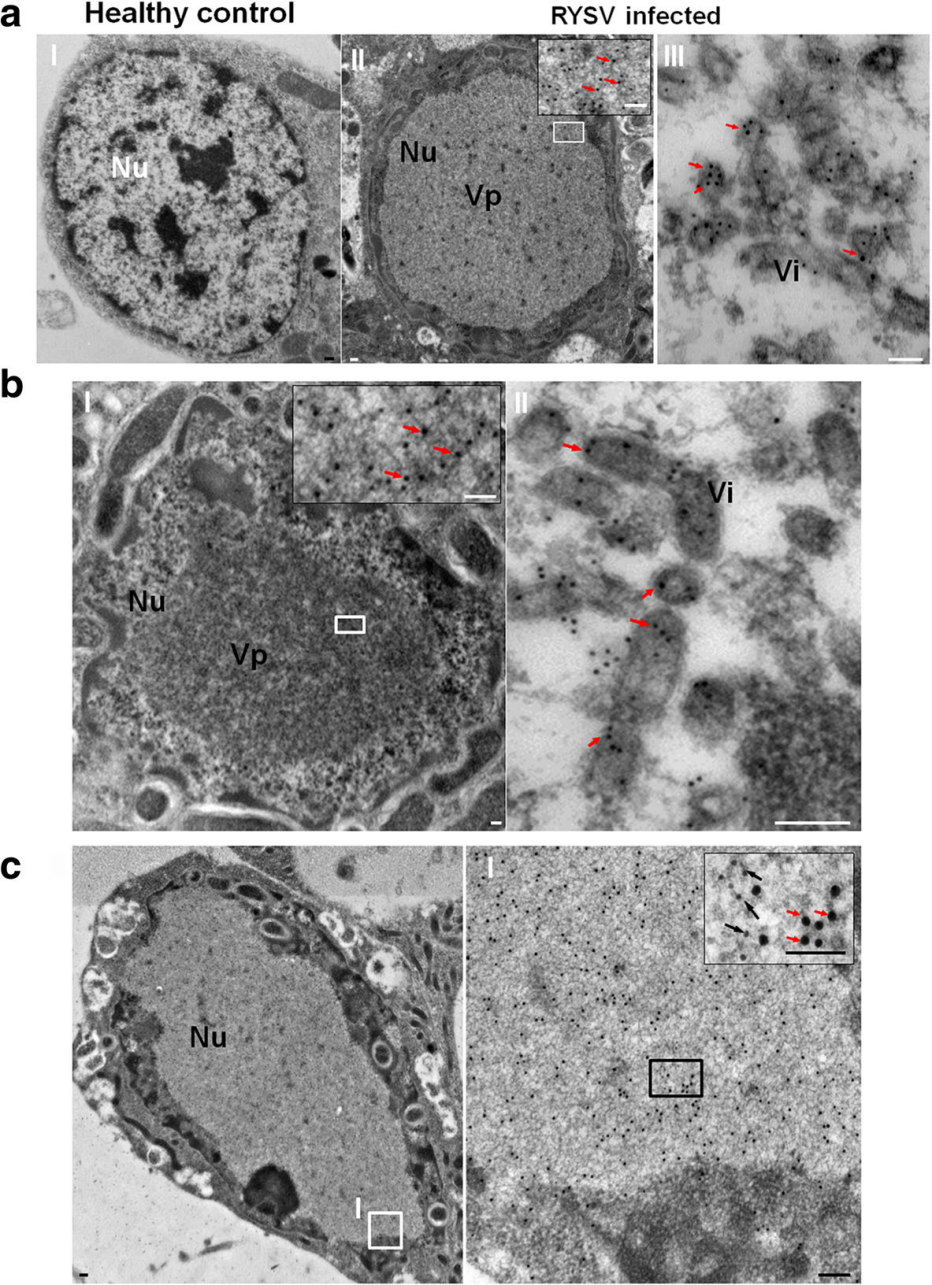
Electron micrographs showing subcellular localization of RYSV structural proteins N and P in RYSV-infected VCMs. **a**, **b** Samples were immunolabeled with (**a**) N- and (**b**) P-specific polyclonal antibodies as primary antibodies, then treated with goat-anti-rabbit IgG conjugated to 15-nm-diameter gold particles as secondary antibodies. **a-I** Healthy cell of VCMs as control; panels (**a-III**) and (**b-II**) are enlarged images of the boxed areas. **c** Double immunogold labeling electron micrographs showing the localization of RYSV structural proteins N and P in RYSV-infected VCMs. Samples were immunolabeled with N-specific rabbit polyclonal antibodies and P-specific mouse polyclonal antibodies as primary antibodies, then treated with goat-anti-rabbit IgG conjugated with 15-nm-diameter gold particles and goat-anti-mouse IgG conjugated with 10-nm-diameter gold particles. Insets are enlarged images of the boxed areas. Black arrows, 10-nm-diameter gold particles; red arrows, 15-nm-diameter gold particles. Nu, nucleus; Vi, virion(s); Vp, viroplasm. Bars, 100 nm

To investigate the composition of the VpLSs formed by RYSV in the nucleus, virus-infected VCMs were fixed at 84 h post inoculation (hpi). Ultrathin sections of RYSV infected VCMs were then subjected to an immunogold labeling assay with the specific rabbit antibodies against N (Fig. 2a II-III) or P (Fig. 2b I-II) as primary antibodies, respectively. This was followed by treatment with goat-anti-rabbit IgG that had been conjugated with 15-nm diameter gold particles as secondary antibodies. Electron microscopic observation showed that N and P antibodies reacted specifically with electron lucent inclusions in the nucleus and the virion (s), respectively (Fig. 2a II-III and b). To determine the composition of these electron-lucent inclusions and further investigate the subcellular relationships of N and P in virus-infected cells, we examined the subcellular localization of both N and P in infected VCMs at 84 hpi using double immunogold electron microscopy with the N (rabbit) and P (mouse) antibodies. As shown in Fig. 2c, both N (15 nm) and P proteins (10 nm) co-localized in the electron-lucent inclusions in the nucleus of infected cell, demonstrating that both N and P proteins were constituents of the VpLSs.

**Fig. 3:**
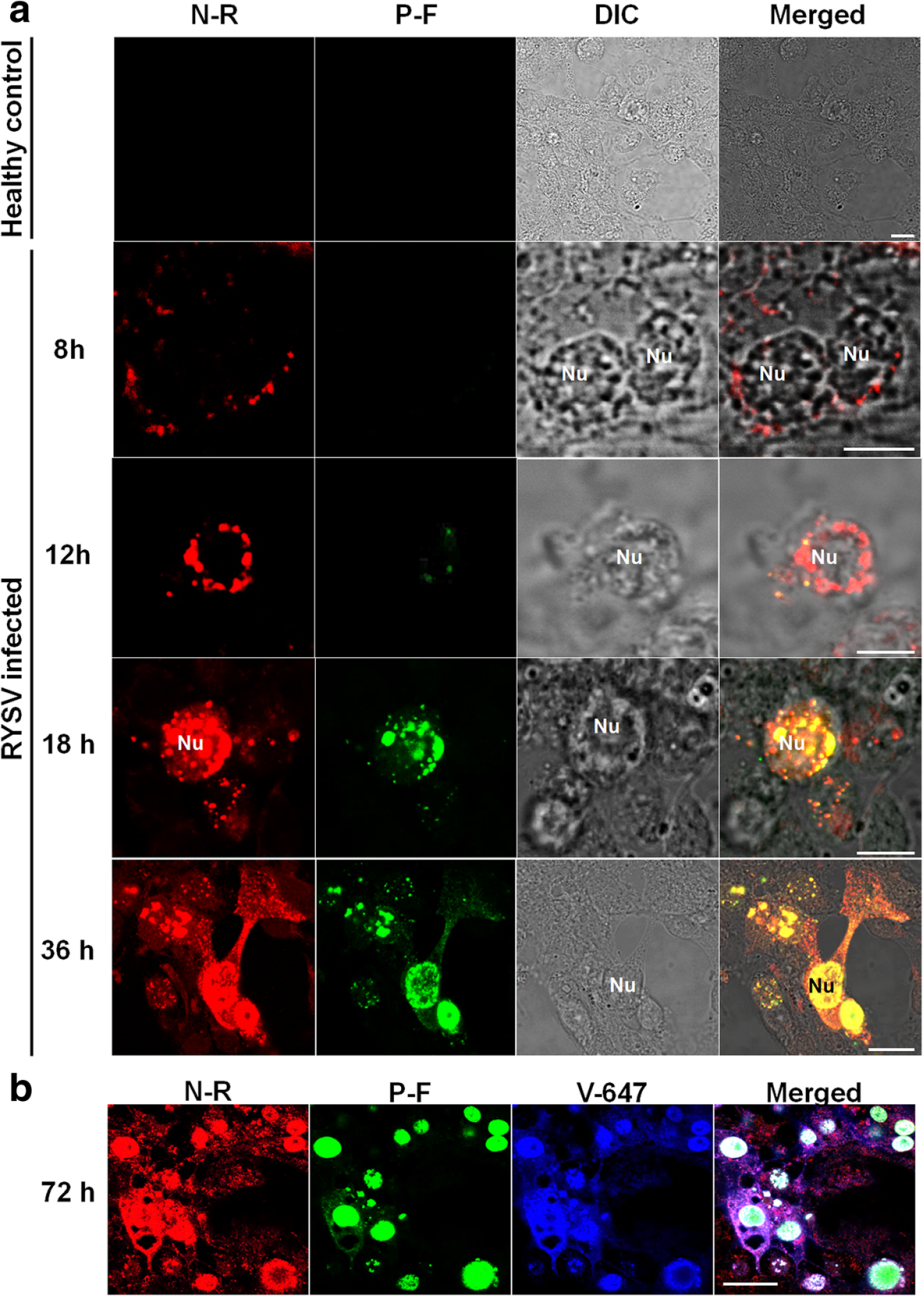
Subcellular localization of N and P of RYSV in viral-infected VCMs. **a** Localization of N and P at different times after RYSV inoculation. Samples were immunolabeled with antibody N-rhodamine (N-R) and P-FITC (P-F). Confocal immunofluorescence micrographs showed that labeling of N before P was imported into the nucleus and that N and P colocalized in the nucleus. **b** Infected VCMs at 72 h after RYSV inoculation and immunolabeled with antibody N-rhodamine (N-R), P-FITC (P-F) and viral-specific IgGs directly conjugated to Alexa 633 (V-633). Bars, 15 μm

To visualize the subcellular localization of structural proteins N and P in virus-infected VCMs, we fixed infected cells at different time points post RYSV inoculation and labeled them with N-specific and P-specific antibodies conjugated directly to fluorescein isothiocyanate and rhodamine, respectively (N-R and P-F) for immunofluorescence microscopy. At 8 hpi, N was first detected in small punctate inclusions in the cytoplasm, and P was undetectable. At 12 hpi, as shown in the third row of Fig. 3a, N surrounded or accumulated in the perinuclear space. The few P protein signals that were initially observed, co-localized with the N protein in the nucleus, but not in the cytoplasm. At 18 hpi, most aggregates formed by N coalesced into large, intensely fluorescing foci in the nucleus and changed remarkably in shape and size, although a few small punctate loci labeled by the N antibody was still observed in the cytoplasm. As shown in the fifth row of Fig. 3a, at 36 hpi, the inclusions formed by N and P gathered into a larger structure, with a morphology similar to that of the viroplasm matrix and filled most of the nuclear space. When the infected VCMs were fixed at 72 hpi and then labeled with N-R, P-F and RYSV virion polyclonal antisera conjugated to Alexa 633, confocal observations showed that structural proteins N and P colocalized with the proteins of RYSV in the nucleus of infected cell (Fig. 3b), suggesting that the nucleus of the infected cell is the site for the replication and assembly of progeny viral particles.

In summary, the above results indicated that the structural proteins N and P were involved in the formation of viroplasm structures in the nucleus of the virus infected insect cell.

### In vitro interactions of RYSV N protein and P protein

**Fig. 4:**
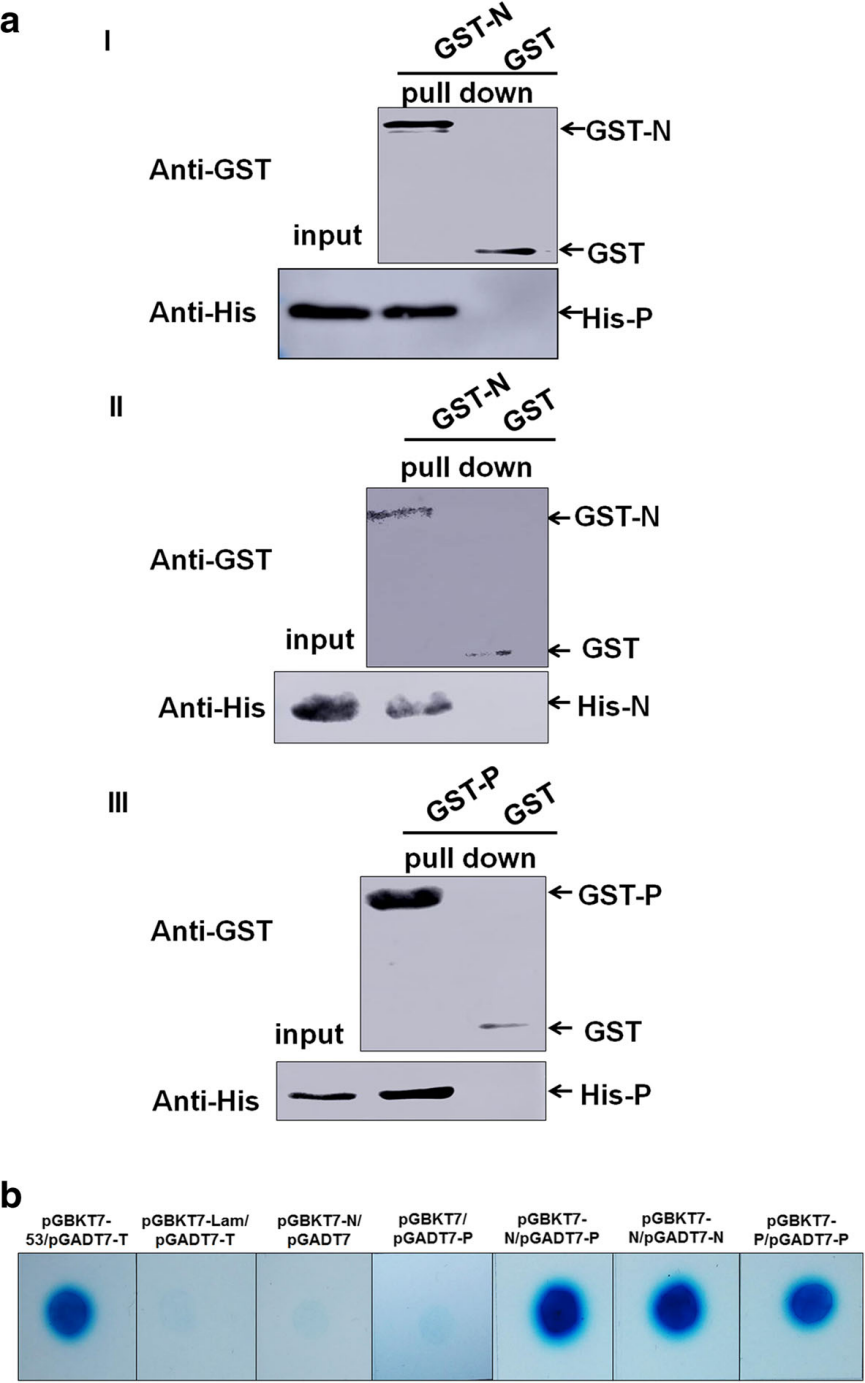
RYSV N specifically interacted with P in vitro. **a-I** Western blot results of GST pull-down assay to detect the interaction of N with P. Bait protein pGEX-3X-N or pGEX-3X was incubated with prey protein pDEST17-P. Antibody against GST was used to detect GST-tagged protein; His antibody was used to detect the bound proteins. The interaction of N with N (**a-II**) and P with P (**a-III**) was also verified by GST pull-down assay. **b** Yeast two-hybrid assay for the interaction of N with P, N with N and P with P. Yeasts transformed with the bait and prey constructs, were grown on SD/−Trp/−Leu/-His/−Ade medium supplemented with 40 μg/ml X-α-Gal. pGBKT7–53 and pGADT7-T co-transformant was used as the positive control. Yeast cells co-transformed with pGBKT7-Lam/ pGADT7-T, pGBKT7-N/pGADT7 and pGBKT7/pGADT7-P were used as negative control

The subcellular colocalization of N and P proteins in the VCMs pointed to a possible interaction between N and P. To substantiate this potential interaction, an in vitro GST pull-down assay was performed. The bacterially-expressed GST-N fusion proteins or GST proteins were immobilized on glutathione beads and incubated with purified His-P protein. Western blotting with antibody to His tag was used to determine the binding of GST-N to His-P. The band positive for His-P was readily detectable in lane containing GST-N, but not in lane containing GST protein (Fig. 4a-I). These results indicated that N interacts directly with P. The self-interaction of N or P is essential for the formation of viroplasm of plant rhabdoviruses, therefore we also detected the interactions of N/N and P/P using the GST pull-down assay. As shown in Fig. 4a II-III, western blotting results shown the self-interaction for both N and P.

When the N and P interactions were investigated in more detail in the yeast two-hybrid assay, as expected, only the yeast cells containing the plasmids pGBKT7–53/pGADT7-T (positive control), pGBKT7-N/pGADT7-P, pGBKT7-N/pGADT7-N and pGBKT7-P/pGADT7-P grew on the SD/−Trp/−Leu/-His/−Ade plate; the negative control groups (pGBKT7-Lam/pGADT7-T, pGBKT7-N/pGADT7, pGBKT7/pGADT7-P) did not. The results of the yeast two-hybrid assay were consistent with that of the pull down experiment and showed that N strongly interacted with P (Fig. 4b). Thus, these results provided convincing in vitro evidences of the interactions of N/P, N/N and P/P.

### N and P proteins are sufficient to form VpLSs in Sf9 cells

**Fig. 5:**
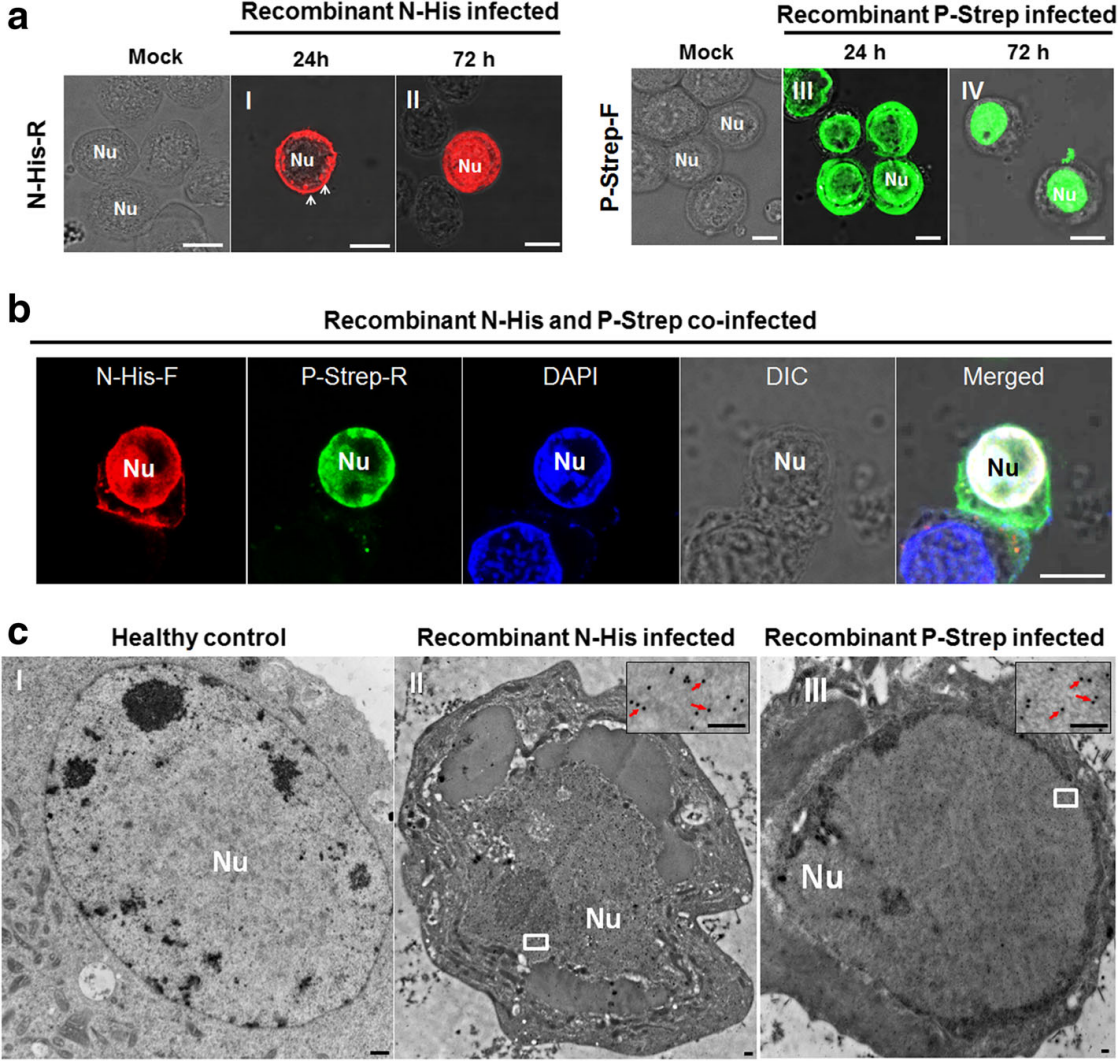
Structural proteins N and P formed viroplasm-like structures in nonhost Sf9 cells. **a** Early and late infection stage of the recombinant protein N-His and P-Strep. **b** Recombinant baculoviruses expressed N-His and P-Strep aggregated in the nucleus and formed viroplasm-like inclusions at 72 hpi. Samples were immunostained with rabbit polyclonal antibodies against His and mouse polyclonal antibodies against Strep, then treated with the corresponding fluorescence-labeled secondary antibody. The nucleus was stained with DAPI. Bars, 10 μm. **c** Electron micrographs showing subcellular localization of fusion proteins N-His and P-Strep at 72 hpi in baculovirus-infected Sf9 cells. I, Healthy non-infected Sf9 cell as control. II, Sf9 cells were infected with baculovirus expressing N-His and immunolabeled with N-specific rabbit polyclonal antibody as the primary antibody; III, cells were infected with baculovirus expressing P-Strep and immunolabeled with P-specific rabbit polyclonal antibodies as the primary antibody. Cells in both II and III were treated with goat-anti-rabbit IgG conjugated to 15-nm-diameter gold particles as the secondary antibody. Insets are enlarged images of the boxed areas. Red arrows, 15-nm-diameter gold particles. Nu, nucleus. Bars, 100 nm

To identify whether N and P possess the inherent ability to form the viroplasm matrix of RYSV, we used the baculovirus expression system to analyze the subcellular distribution of N and P in Sf9 cells. The N and P genes were cloned into the baculovirus expression plasmid encoding the fusion protein with 6 × His tag and a Strep tag, respectively. Each recombinant baculovirus was then used to separately infect Sf9 cells. As observed via immunofluorescence microscopy, at 24 hpi, the transiently expressed N protein self-assembled in the cytoplasm around the nucleus into small aggregates (Fig. 5a I), which gradually migrated into the nucleus and spread throughout the cell at 72 hpi (Fig. 5a II). In contrast to the distribution of the N protein, when expressed individually, the P-strep was scattered diffusely throughout the cells and did not assemble into any specific structures at an early stage of recombinant baculovirus infection (Fig. 5a III). Strikingly, at 72 hpi, the fusion protein P-Strep had largely accumulated in the nucleus of Sf9 cell (Fig. 5a IV). When the recombinant baculovirus expressing N or P were used to co-infect the Sf9 cells, at 72 hpi, the N fusion protein had colocalized with the P fusion protein only in the nucleus (Fig. 5b). To further study the structures formed by N-His and P-Strep in the nonhost Sf9 cells at 72 hpi, we used immunogold electron microscopy to localize the fusion proteins N and P. As shown in Fig. 5c, in the nucleus, N and P antibodies reacted specifically with the VpLSs which appeared similar to the viroplasm structures observed in RYSV-infected VCMs. Thus, N colocalized with P proteins and resulted in the formation of VpLSs in the nucleus of the nonhost Sf9 cell.

### RNAi induced by dsN and dsP inhibited the infection of RYSV in VCMs

**Fig. 6:**
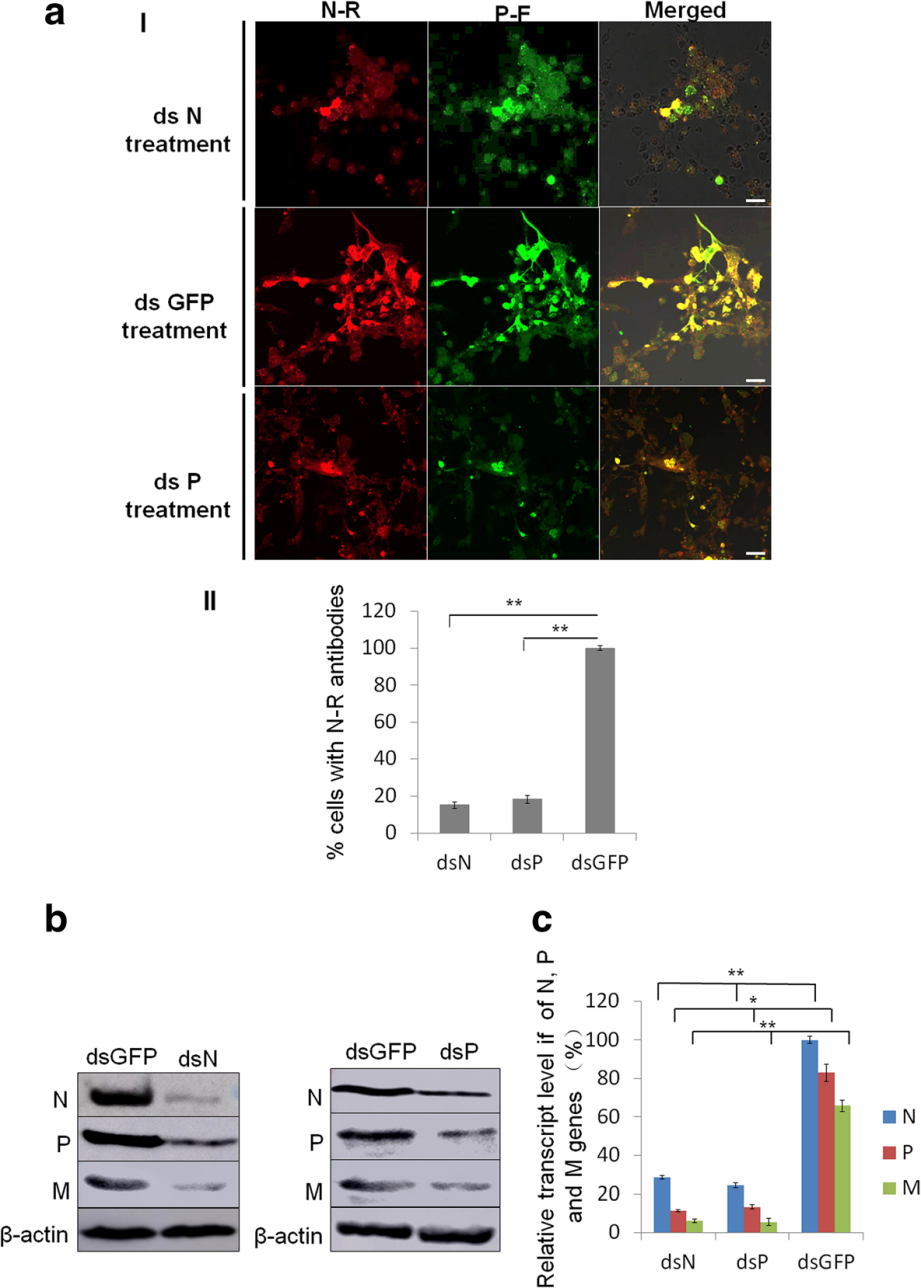
RNAi induced by double-stranded RNAs *dsN* and *dsP* inhibited RYSV infection of VCMs. **a-I** VCMs were transfected with *dsN*, *dsP* or *dsGFP* and then inoculated with RYSV. At 72 h post RYSV inoculation, cells were immunolabeled with N-R and P-F, and examined with a confocal microscope. Bars, 10 μm. **a-II** Quantitative analysis of RYSV infected cultured cells was indicated with N-R antibodies by confocal microscopy at 72 h post RYSV inoculation (1200 cells/condition were counted, mean ± SD; **, *p* < 0.01). **b** Western blots of viral proteins from VCMs transfected with dsRNAs. N-, P- and M-specific antibody were used to detect N, P and M protein, respectively, at 84 hpi. Actin was used as the control and detected with β-actin-specific antibody. **c** Relative transcript levels of N, P and M genes for each treatment determined by qPCR. Three biological repeats were done. Data represent means ± SD and were analyzed using Student’s *t*-test; significance levels: *p < 0.05; **p < 0.01

To investigate the functional role of RYSV structural proteins N and P during the early process of viral infection in VCMs, cells were transfected *dsN*, *dsP* and *dsGFP*, respectively, then incubated with the RYSV inoculum. At 72 hpi, the infected VCMs with different dsRNA treatments were immunolabeled with specific antibodies against N and P, N-Rhodamine (N-R) and P-FITC (P-F). Confocal microscopy observation showed that far fewer inclusion bodies were formed by N and P in the cells transfected with *dsN* or *dsP*, (Fig. 6a-I, a-II). In contrast, abundant N and P proteins accumulated in the nucleus of the RYSV-infected cells treated with *dsGFP* (Fig. 6a). These results confirmed that both N and P proteins play important roles in the establishment of the viroplasm.

The effects of RNAi induced by dsRNAs on the viroplasm formed by N and P at 84 hpi were further assessed using western blots with N-, P- and matrix protein M-specific antibodies to detect the level of the corresponding proteins (β-actin served as the control). As expected, the accumulation of N and P decreased significantly in VCMs treated with either *dsN* or *dsP*, and the accumulation of M protein was also reduced when the N or P gene was knocked down (Fig. 6b). This result was confirmed by RT-qPCR. The transcript levels of N, P and M genes after *dsN* and *dsP* treatment were reduced significantly compared with the *dsGFP* treatment in RYSV-infected VCMs. All together, these results suggest that the knockdown of N and P genes impaired the assembly of the viroplasm, which would thus reduce accumulation of RYSV (Fig. 6c).

## Discussion

Among the viruses in the *Rhabdoviridae* family, members of the genus *Nucleorhabdovirus* are believed to assemble their viroplasm and multiply in the nucleus of cell derived from both their host plants and insect vectors [2, 3]. The replication strategy of nucleorhabdoviruses has been well documented in previous studies in host plants, but not in the insect vector. Although PYDV infection of leafhopper cell cultures has been well studied, the detailed assembly of its viroplasm in insect cells has not been reported so far [33]. The present study, taking advantage of the cultured leafhopper cell line, is the first report of the cellular location and morphogenesis of viroplasms of RYSV in insect vector cells.

Our electron microscope observations provide evidence that during infection in insect cells, RYSV executed a replication strategy similar to those of other nucleorhabdoviruses in plant cells, namely producing N and P to form an intranuclear electron-lucent viroplasm (Figs. 1 and 2). The unenveloped RYSV virions were assembled and accumulated in the periphery of the viroplasm inside the nucleus. Like in plant cells, nucleocapsids interact with the nuclear membrane and are then transported into the cytoplasm in a budding process. Most mature RYSV virions with a host-derived lipid envelope were scattered around the periphery of the nucleus, while few accumulated in the perinuclear space (Fig. 1). In addition, the nucleus became enlarged after the assembly of the viroplasms in the infected cells (Figs. 1 and 2), and the morphological changes in the nucleus that RYSV induced were consistent with PYDV and SYNV viroplasm formation in plant cells [34, 35]. Immunofluorescence observation of RYSV-infected cells showed that early in RYSV infection, the N protein was expressed before P and formed small aggregates in cytoplasm around the nucleus before being transported into the nucleus. Curiously, the interaction between N and P occurred exclusively inside the nucleus of infected cell after both proteins were imported into the nucleus. A similar strategy was also reported for PYDV and SYNV [7, 9, 36].

We further investigated the morphological variation of N and P in the model insect cell line Sf9 using a baculovirus system. Results from confocal and electron microscopy of N and P expressed in nonhost Sf9 cells, clearly indicated that when the proteins were separately expressed by the recombinant baculovirus vector, N was generally distributed throughout the nucleus, while P was entirely localized in the nucleus. However, when P and N were coexpressed, both proteins were recruited to a subnuclear region to induce a large viroplasm-like focus that was similar to the viroplasm induced by RYSV in the vector *N. cincticeps* cells. These results suggested a strong physical interaction between N and P proteins, which was subsequently confirmed by the GST pull-down assay and yeast two- hybrid assay. This interaction between N and P seems to be a common phenomenon in the plant and animal rhabdoviruses [37, 38].

Several key issues in the replication mechanism of plant rhabdoviruses remain unexplored in their insect vectors. For example, the mechanisms by which RYSV N and P proteins are imported into the nucleus are still unknown. The nucleorhabdovirus-encoded N and P are believed to be imported into the nucleus through an importin α/β dependent pathway. N of SYNV and PYDV was proved to contain NLSs, which interact with importin-α and are responsible for the nuclear import of N [37]. Although the previous studies suggested that N of RYSV may possess NLSs [39], whether RYSV N is imported through the same strategy as SYNV and PYDV or through the participation of host components needs further study. P proteins of SYNV were proved to contain NLSs, but its entry into the nucleus rely on an importin-independent pathway [37]. However, an investigation of orchid fleck virus (OFV), another plant rhabdovirus, showed that the P proteins, but not N proteins have NLSs and interact with importin α1 and importin α2 in *Nicotiana benthamiana* [40]. Whether there is a similar interaction between P proteins of RYSV and a vector leafhopper importin α protein also needs to be investigated.

## Conclusion

In this study, we present immunofluorescence and electron microscopic evidence for the morphogenesis of RYSV viroplasm in its insect cell. We identified the cellular localization during viral infection and determined the interaction between N and P proteins in vivo and in vitro. Finally, we showed that knocking down the RYSV transcription of N and P significantly inhibited the infection of RYSV in leafhopper culture cells. This is the first report for the precise delineation of the roles of the structural proteins in a nucleorhabdovirus involved in the assembly of viroplasm in insect cell cultures.

## Additional file

Additional file 1: Sequences of the forward primer and reverse primer for RYSV N, P, M and *Nephotettix cincticeps* actin genes. (PDF 10 kb)
