# Supplementary material for: Development of leafhopper cell culture to trace the early infection process of a nucleorhabdovirus, rice yellow stunt virus, in insect vector cells
Source: Virol J. 2018 Apr 20;15:72. doi: 10.1186/s12985-018-0987-6 (PMC5910589; doi:10.1186/s12985-018-0987-6)
Supplement: Supplementary file 1 — Sequences of the forward primer and reverse primer for RYSV N, P, M and Nephotettix cincticeps actin genes. (PDF 10 kb) [file 12985_2018_987_MOESM1_ESM.pdf]

Primer name	Primer Sequence
qPCR-RYSV-N-F	AGTATGCCCAACTTGCCAGG
qPCR-RYSV-N-R	CATTCGTTCAACCGGCATCC
qPCR-RYSV-P-F	CTTTAACAGGGGTGGCAAGC
qPCR-RYSV-P-R	TTGGGACGTTTTGCTCCAGT
qPCR-RYSV-M-F	CCCGATCATGAAGCCACTAC
qPCR-RYSV-M-R	CTTATTGTAGCACCCACCCC
qPCR-Nc-Actin-F	GGGATACAGTTTCACCACG
qPCR-Nc-Actin-R	GACACCTGAATCGCTCGT
